# Real‐Time PCR *Helicobacter pylori* Test in Comparison With Culture and Histology for *Helicobacter pylori* Detection and Identification of Resistance to Clarithromycin: A Single‐Center Real‐Life Study

**DOI:** 10.1111/hel.70031

**Published:** 2025-04-07

**Authors:** Kateryna Priadko, Sophie‐Anne Gibaud, Amaury Druet, Louise Galmiche, Francis Megraud, Stéphane Corvec, Tamara Matysiak‐Budnik

**Affiliations:** ^1^ Nantes Université, CHU Nantes, IMAD, Hepato‐Gastroenterology & Digestive Oncology Nantes France; ^2^ Department of Bacteriology and Microbiological Control University Hospital of Nantes Nantes France; ^3^ Department of Pathology University Hospital of Nantes Nantes France; ^4^ INSERM U1312 BRIC, University of Bordeaux Bordeaux France; ^5^ INSERM, Immunology and New Concepts in ImmunoTherapy, INCIT, UMR 1302 Nantes Université Nantes France

**Keywords:** clarithromycin resistance, culture, gastritis, *Helicobacter pylori*, histology, RT‐PCR

## Abstract

**Background:**

In our center, RT‐PCR was integrated as a routine method to diagnose *Helicobacter pylori* due to its higher availability after COVID‐19 pandemics. The objective of this study was to assess the feasibility and performance of systematically performed RT‐PCR for *H. pylori* detection and clarithromycin (CLA) resistance in a real‐life clinical practice.

**Materials and Methods:**

One hundred consecutive patients underwent an upper digestive endoscopy during which at least four biopsies (two from the antrum and two from the corpus) were obtained for RT‐PCR and culture with antibiogram and four additional biopsies for histology. The results of *H. pylori* detection were compared among RT‐PCR, histology, and bacterial culture, and the results of CLA susceptibility were compared between culture‐based antibiogram and RT‐PCR.

**Results:**

Out of 100 patients, 64 were positive for *H. pylori* by RT‐PCR, 66 by histology, and 53 by culture, with no statistically significant difference among the three methods (*p* > 0.05). CLA resistance was found in 8 out of 45 patients (17.7%) by culture and in 12 out of 64 patients (18.7%) by PCR. In 8 *H. pylori* ‐positive patients by culture, the antibiogram could not be realized due to lack of viability of the strains. In one patient, after a double checking, discrepant results were observed, requiring a complementary molecular analysis by the French National Reference Center for Helicobacters, which confirmed the existence of a double population of *H. pylori* strains within biopsies, with and without CLA resistance.

**Conclusions:**

Our study demonstrates that in real‐life clinical practice, RT‐PCR is feasible and comparable in the ability to detect *H. pylori* and its resistance to CLA to bacterial culture with antibiogram and histology. Given its rapidity and limited dependence on the operator's interpretation, it appears preferable to the other methods.

## 1 Introduction

*Helicobacter pylori* is a Gram‐negative bacterium that infects about half of the world's population, inducing chronic gastritis, now formally recognized as an infectious disease. More importantly, chronic *H. pylori* ‐associated gastritis may progress to severe complications such as peptic ulcer disease, gastric adenocarcinoma, and gastric MALT lymphoma [1]. *H. pylori* prevalence declined from 58.2% in the 1980–90 period to 43.1% in the 2011–22 period, with the largest decline observed in the Western Pacific, Southeast Asian, and African regions [2, 3]. This decrease in *H. pylori* prevalence has been associated with a global decrease in gastric cancer incidence in the same areas [3].

According to the European Registry (HpEuReg) database, the initial diagnosis of *H. pylori* infection is most commonly established by invasive tests (71%), less frequently by non‐invasive tests (41%), and by both types of tests only in 12% of the cases. Up to date, the most common invasive test used for *H. pylori* detection in Europe is histology [4]. The choice of the most appropriate *H. pylori* diagnostic test depends on the specific individual patient history and local availability [5]. Another factor to consider in the choice of the initial diagnostic test for *H. pylori* infection is the antimicrobial susceptibility given the emergence of resistant strains, especially to clarithromycin (CLA), due mainly to the use of this antibiotic to treat respiratory tract infections. A tailored antimicrobial treatment based on the results of antibiotic susceptibility testing (AST) is then highly encouraged in order to overcome the issue of empirical therapy failure and avoid increased antibiotic resistance worldwide [1, 6, 7].

RT‐PCR has been recognized for its high sensitivity and specificity in the detection of pathogens, including *H. pylori* [8, 9, 10]. In contrast, the COVID‐19 pandemic made this technique more accessible due to the availability of thermocyclers in clinical establishments of all levels.

In this study, we aimed to analyze the performance and feasibility of the commercially available kit RIDAGENE *H. pylori* RT‐PCR (R‐Biopharm AG, Darmstadt, Germany) in routine clinical practice and comparing it with conventional tests of culture with antibiogram in their ability to detect *H. pylori* and its resistance to CLA on gastric biopsy specimens. Indeed, this method has shown excellent results in previous studies [11].

Furthermore, we performed histologic analysis of all the biopsy specimens and analyzed the association between *H. pylori* status and histological findings.

## 2 Materials and Methods

In this “real‐life” study, all the consecutive patients referred to the University Hospital of Nantes in the time period between September 2021 and December 2023, for *H. pylori* eradication after one or more eradication failures, were included. According to standard procedure in our center, all these patients underwent an upper gastrointestinal endoscopy (UGE) with multiple gastric biopsies for AST, and in particular sensitivity to CLA, in order to receive the next line of treatment based on these results.

The study was performed according to the Declaration of Helsinki and was approved by the Institutional Ethical Committee. All patients signed an informed consent for the procedure as well as for participation in the study.

During the UGE, four biopsies (two antral and two corpus) were obtained for RT‐PCR and culture, followed subsequently by four gastric biopsies (two from the antrum and two from the corpus) obtained for histology. Biopsies for RT‐PCR and culture were introduced in a special transport medium (Portagerm, bioMérieux, Craponne, France) and kept at 4°C before being transported rapidly to the Bacteriology Department. Biopsies for histology were introduced in a fixative and sent to the Department of Pathology.

### 2.1 Treatment of the Biopsies

After arrival to the Bacteriology Department, the four gastric biopsies were crushed together in a single 1.5 mL tube containing 500 μL of brain heart infusion broth (bioMérieux) with a sterile grinder. A few drops of the ground material were immediately inoculated onto the agar plates warmed to room temperature for culture, while the remaining material was frozen and stored at −80°C for PCR.

### 2.2 Culture

Agar plates were incubated immediately at 35°C ± 2°C in a microaerobic atmosphere that was renewed every 48 h. Monitoring of the inoculated plates took place every 48 h for 12 days. The small, shiny, round, and domed colonies suspected to be *H. pylori* were confirmed by microscopic examination and biochemical tests.

### 2.3 Antibiotic Susceptibility Testing

When *H. pylori* detection was positive, an antibiogram was performed according to the EUCAST recommended method [12, 13], a McFarland 3 suspension was inoculated on a Muller‐Hinton agar plate, and the following antibiotics were tested: CLA, amoxicillin, levofloxacin, and rifampicin by using gradient concentration E‐tests. Plates were incubated for 48 h at 37°C in a microaerobic atmosphere. EUCAST criteria were used to determine susceptibility or resistance.

### 2.4 Real‐Time PCR

The technique used was a real‐time PCR (RT‐PCR) using the RIDAGENE *H. pylori* RT‐PCR kit, performed on the BDMax automated PCR system (Becton‐Dickinson, Le Pont de Claix, France). The tests were performed according to the manufacturer's instructions.

CLA resistance for *H. pylori* is only caused by a limited number of mutations in the 23S rDNA at positions 2142 and 2143, all detected by the kits used.

### 2.5 Histology

Gastric biopsies were fixed in 10% phosphate‐buffered formalin and embedded in paraffin. Serial sections of 3 μm were made for hematoxylin‐eosin (H&E) staining and histopathological evaluation, and for immunohistochemical examination (IHC) in selected cases. The IHC technique was performed using an automated Dako Omnis automated system (Agilent Technologies, Santa Clara, CA, USA), using the streptavidin‐biotin amplification technique after appropriate antigen retrieval.

Slides were stained against *H. pylori* (prediluted; Agilent polyclonal). The peroxidase activity was revealed using 3,30‐diaminobenzidine for 5 min. The slides were then counterstained with hematoxylin.

*H. pylori* IHC was performed when no *H. pylori* was observed on standard staining despite concomitant chronic gastritis, focal gastritis, or doubtful standard staining and control endoscopy after *H. pylori* eradication.

### 2.6 Statistical Analysis

All measured variables were analyzed by descriptive statistics. We included the categorical variables providing sample size for RT‐PCR, culture, and histology. Sensitivity, specificity, their 95% confidence intervals (CIs), and *p*‐value were calculated using STAT plus application Basic version for Windows.

## 3 Results

### 3.1 Patients

One hundred patients (mean age 49.9 years) underwent upper GI tract endoscopy procedure with at least four biopsies (two antral and two corpus) for culture with antibiogram and RT‐PCR and four biopsies for histology. Among all the patients, 57% were immigrants from African regions as well as from Eastern Europe.

### 3.2 *Helicobacter pylori* Detection

Among 100 patients, 64 were found to be *H. pylori* ‐positive by PCR, 53 by culture, and 66 by histology (Table 1). Histology showed 100% sensitivity and specificity when compared to the gold standard that was established as histology and/or PCR positive.

**TABLE 1:** Results of *Helicobacter pylori* diagnosis and its resistance to clarithromycin performed by RT‐PCR, culture with antibiotic susceptibility testing, and histology.

Test	*N* (%)	Sensitivity, % (95% CI)	Specificity, %, (95% CI)	*p*
*H. pylori* PCR positivity	64/100 (64%)	96.97% (89.48%–99.63%)	100% (89.72%–100.00%)	*p* > 0.05
CLA resistance by PCR	12/64 (18.7%)	100% (66.37%–100.00%)	100% (90.26%–100.00%)	
*H. pylori* culture positivity	53/100 (53%)	80.30% (68.68%–89.07%)	100% (89.72%–100.00%)	
CLA resistance by antibiogram	8/45 (17.7%)	90% (55.50%–99.75%)	100% (90.26%–100.00%)	
*H. pylori* histology positivity	66/100 (66%)	100% (94.56%–100.00%)	100% (89.72%–100.00%)	

Abbreviations: AST, antibiotic susceptibility testing; CI, confidence interval; CLA, clarithromycin; *H. pylori* , *Helicobacter pylori* .

The sensitivity of PCR and culture was 96.9% (95% CI 89.48%–99.63%) and 80.30% (95% CI 68.68%–89.07%), respectively, and specificity was 100% for both.

In two patients with discrepant results between PCR and histology (histology positive while PCR negative), IHC was performed on the gastric biopsies to confirm the presence of *H. pylori* . It confirmed that P#1 was negative on antral biopsies, but rare *H. pylori* were found in fundic biopsy samples. In this patient, macroscopically, the gastric mucosa was normal at endoscopy, but microscopically, a reactive chronic gastritis in the antrum and a slightly active chronic gastritis in the fundus were found. In P#2, with discrepant results, the inverse situation was observed: in the antral biopsies, rare *H. pylori* were found, while fundic biopsies were negative. Microscopically, the samples exhibited light to moderate antral gastritis. It should be highlighted that in the samples with negative *H. pylori* by PCR and culture, histology found only very rare *H. pylori* . Most likely, the quantity of bacteria was under the detection threshold by PCR.

### 3.3 Clarithromycin Resistance Detection

For the detection of CLA resistance, PCR showed 100% sensitivity and specificity when compared to the gold standard that was established as PCR and/or antibiogram positive for resistance. The sensitivity and specificity of CLA resistance by antibiogram when compared to the gold standard were 90% (95% CI 55.50%–99.75%) and 100%, respectively (Table 1).

Among 64 RT‐PCR‐positive samples, mutations associated with CLA resistance were detected in 12 samples (18.7%). Antibiogram was performed only on 45 out of 53 *H. pylori* ‐positive samples by culture due to the lack of viability of culture material from five samples and technical issues for three samples. CLA resistance was detected in 8/45 (17.7%) samples (Table 1). All these eight samples were also CLA resistant by PCR. No statistically significant difference was observed between the two tests in their ability to detect *H. pylori* or its resistance to CLA (*p* > 0.05).

In one patient, a discrepancy of the results between PCR and culture was observed. Both tests were positive for *H. pylori* ; however, while PCR showed CLA resistance, the antibiogram showed sensitivity to CLA. This finding was confirmed by molecular analysis performed at the National Reference Center for Helicobacters, indicating the existence of the double population of *H. pylori* , with and without resistance to CLA (i.e., heteroresistance).

### 3.4 Microscopic Analysis of Biopsy Samples

**FIGURE 1:**
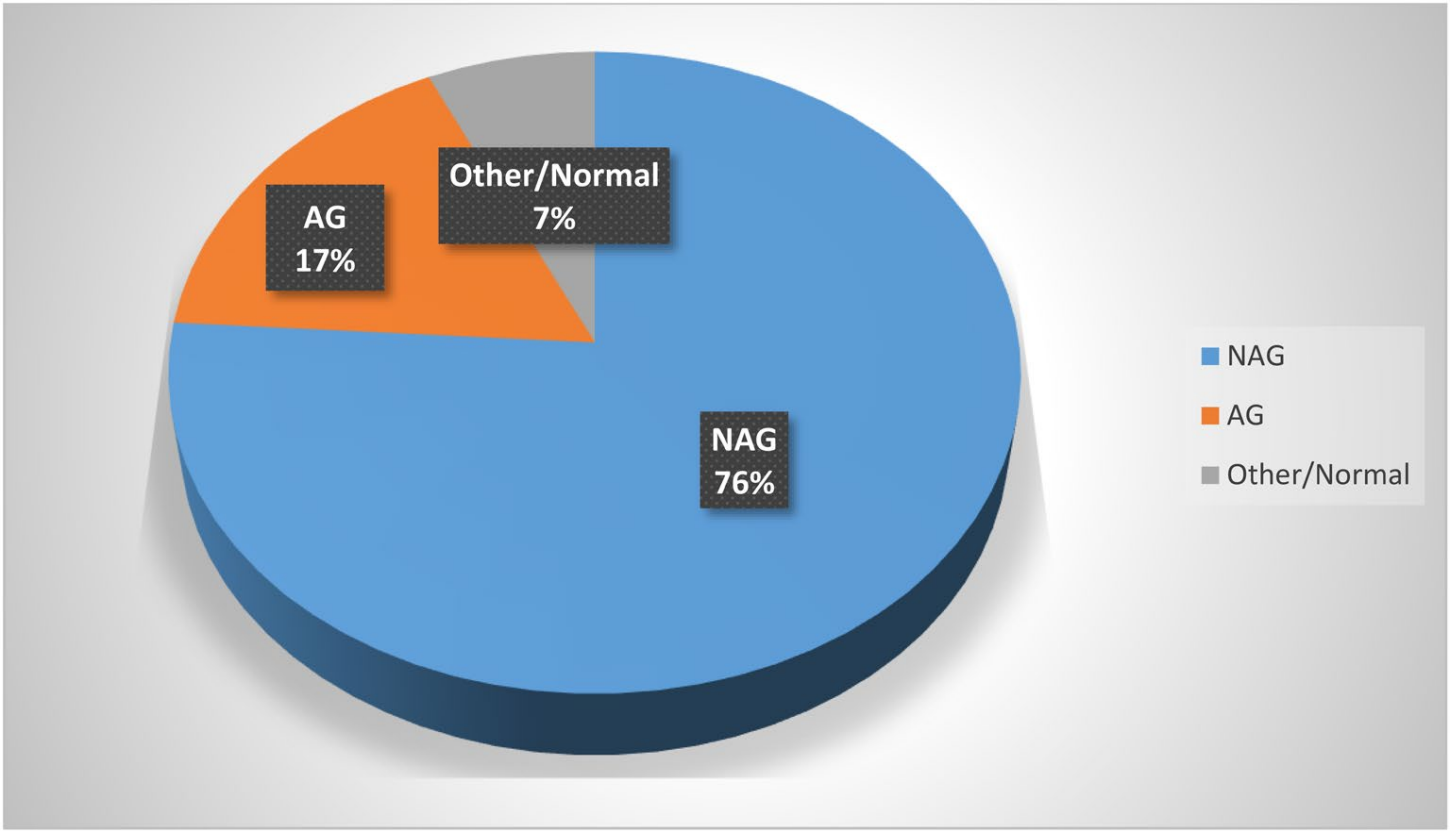
Microscopic findings in our population. AG, atrophic gastritis; NAG, non‐atrophic gastritis.

Among 100 patients, histologic evaluation of gastric biopsies indicated 76 cases with non‐atrophic gastritis (NAG), 17 with atrophic gastritis (AG), and 7 with other findings or normal histology (Figure 1).

**FIGURE 2:**
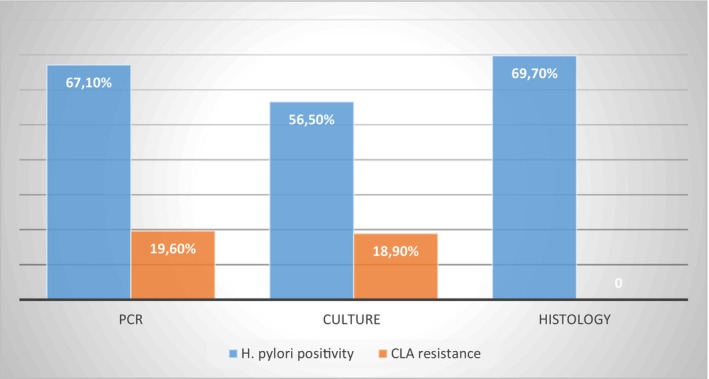
*Helicobacter pylori* (blue) and clarithromycin resistance (orange) positivity rate in patients with non‐atrophic gastritis using different tests. CLA, clarithromycin; *H. pylori* , *Helicobacter pylori* .

Among NAG cases (*N* = 76), 67.1% (51/76) were *H. pylori*‐positive by PCR, 56.5% (43/76) positive by culture, and 69.7% (53/76) positive by histology. CLA sensitivity among NAG was as follows: 7 CLA resistant by antibiogram (18.9%) and 10 CLA resistant by PCR (19.6%) (Figure 2). The two cases where histology showed superiority over PCR in *H. pylori* detection were described as normal (P#1) and as antral gastritis (P#2) by histological evaluation.

**FIGURE 3:**
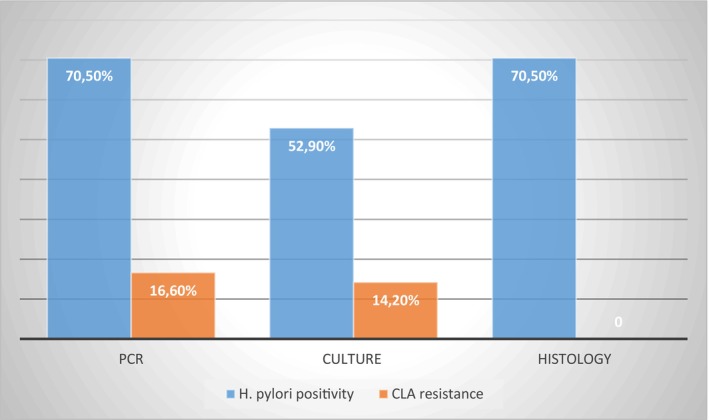
*Helicobacter pylori* (blue) and clarithromycin resistance (orange) positivity rate in patients with atrophic gastritis using different tests. CLA, clarithromycin; *H. pylori* , *Helicobacter pylori* .

Among the patients with AG (*N* = 17), 12 were *H. pylori* ‐positive by PCR (70.5%), 9 by culture (52.9%), and 12 by histology (70.5%). One sample was CLA resistant by antibiogram (14.2%) and two by PCR (16.6%) (Figure 3).

For two *H. pylori*‐positive patients by culture with AG and six with NAG, the antibiogram could not be performed due to the lack of viability of the material or technical issues.

Among the patients with AG, some cases with a discrepancy between PCR and culture were observed, PCR being superior to culture but equal to histology for *H. pylori* detection: P#1—moderate atrophy and intestinal metaplasia (IM) in fundus, P#2—chronic atrophic pangastritis with severe IM (OLGA 4 and OLGIM 4) and P#3—suspicion of gastric MALT lymphoma. In all these patients, both PCR and histology were positive while culture was negative for *H. pylori* .

## 4 Discussion

In our real‐life study, we confirm that RT‐PCR is feasible, has excellent sensitivity, is less time‐consuming, can be performed in a few hours compared to several days for culture & antibiogram, and is less dependent on the experience of the operator when compared to other conventional *H. pylori* testing methods. Moreover, the results of *H. pylori* identification by RT‐PCR are comparable to histology and culture, which is in line with previously published data [10, 14, 15].

Also, RT‐PCR's ability to detect *H. pylori* resistance to CLA was shown to be superior over culture, especially in patients with moderate to severe AG and suspicion of MALT lymphoma. Culture with antibiogram used to be a gold standard in *H. pylori* identification and CLA resistance detection; however, the method presents some limits for routine clinical use: it is relatively expensive, more complicated, and time‐consuming as it requires an incubation for 5–7 days at 37°C [16]. In our study, culture was shown to be the least sensitive in *H. pylori* detection. Moreover, in 8 out of 53 *H. pylori* ‐positive by culture, an antibiogram could not be performed due to lack of viability of the material or technical issues. Hence, we believe that culture with an antibiogram remains a useful tool in clinical practice only in cases of CLA‐resistant strains because it allows testing the susceptibility to all the other antibiotics of potential use [16].

Even though PCR is usually considered superior to histology for the detection of *H. pylori* [17, 18, 19], the particularly good performance of *H. pylori* detection by histology observed in our study may be related to the high number of biopsies obtained (at least 4), which corresponds to the standard of clinical management in our center considered an expert center. Indeed, it has been reported previously that the number of biopsies correlates with the probability of *H. pylori* detection [20, 21, 22].

We did not find a difference in *H. pylori* positivity rate between the patients with AG and NAG, while a lower rate of *H. pylori* positivity in AG is frequently described. This result may also be partly explained by the relatively high number of biopsies examined, thus allowing us to detect the bacteria even if their number is decreased.

The higher detection rate of *H. pylori* by RT‐PCT over culture (70.5% vs. 52.9%) in AG may be explained by the higher sensitivity of RT‐PCT in the conditions of decreased bacterial load in atrophic mucosa [23].

The patient's demography such as age and ethnic origins may affect the prevalence of *H. pylori* . Current higher prevalence of the infection in France is mostly found among recent immigrants from countries with high prevalence, that is, Africa and Europe, Portugal, and some East European countries. Indeed, in our cohort, 57% of patients were of foreign origin, in particular North African and East European.

Our study has some limitations, including its single‐center design, relatively small sample size, reduced sensitivity of the RT‐PCR technique to low bacterial loads, and the lack of cost‐effectiveness analysis that may be responsible for some biases and do not allow generalization of the results and extrapolation to other settings and populations.

In this pragmatic, “real‐life” study, we aimed to propose the simplest strategy that might be useful in daily clinical practice, and in particular, highlighting the usefulness of PCR, which can test only CLA resistance that is crucial for treatment choice in France (optimized triple therapy may be prescribed in case of sensitive strains). The resistance to other antibiotics is much less important in a clinical setting: very few resistant strains to amoxicillin, tetracycline, or rifabutin, and the resistance of limited clinical importance to metronidazole. For levofloxacin, it can be interesting to know, and there is a test commercially available, but this antibiotic should be used as little as possible following the recent recommendations linked to its adverse events.

The major value of our study is that it highlights a practical point that in France, in the case of CLA‐sensitive strains, the patients can receive an optimized triple therapy according to the stewardship principles, which limit the adverse events as compared to the bismuth quadruple therapy and avoid having a potentially unnecessary antibiotic like with the concomitant therapy [24].

However, larger multicenter, prospective studies are needed to confirm the value of AST, including additional resistance genes, as well as other non‐invasive tests, like stool‐based PCR, in clinical practice and management of the patients, and taking into account the cost‐effectiveness of this approach.

## 5 Conclusions

We conclude that RT‐PCR in *H. pylori* detection was highly convenient, the fastest among all tests, and can be recommended for routine practice, while culture should be kept to test the susceptibility to other antibiotics in case of CLA‐resistant strains.

## Author Contributions

K.P., S.‐A.G., and T.M.‐B.: Study design, data collection, and results analysis. A.D.: Data collection and analysis. L.G.: Histological analysis. S.‐A.G. and S.C.: PCR analysis. K.P.: Manuscript drafting. F.M.: Critical review of the manuscript. All: Manuscripts review and approval.

## Conflicts of Interest

The authors declare no conflicts of interest.

## Data Availability

The data that support the findings of this study are available from the corresponding author upon reasonable request.
